# Antibiotic Consumption in Primary Care in Costa Rica and Italy: A Retrospective Cross-Country Analysis

**DOI:** 10.7759/cureus.41414

**Published:** 2023-07-05

**Authors:** Esteban Zavaleta, Francesco Ferrara, Andrea Zovi, José Pablo Díaz-Madriz, Abigail Fallas-Mora, Bruno Serrano-Arias, Filomena Valentino, Sebastián Arguedas-Chacón, Roberto Langella, Ugo Trama, Eduardo Nava

**Affiliations:** 1 Pharmacy, Hospital Clinica Biblica, San Jose, CRI; 2 Pharmacy, Asl Napoli 3 Sud, Napoli, ITA; 3 Pharmacy, Ministry of Health, Rome, ITA; 4 Pharmacy, Hospital Clínica Bíblica, San José, CRI; 5 Pharmacology and Therapeutics, Hospital Clinica Biblica, San Jose, CRI; 6 Therapeutics, Hospital Clinica Biblica, San Jose, CRI; 7 Therapeutics, University of Milan, Milan, ITA; 8 Pharmacy, Ministry of Health, Milano, ITA; 9 Pharmacy, Asl Napoli Sud, Nola, ITA

**Keywords:** awareness campaigns, antibiotic stewardship programs, antibiotic expenditure, antibiotic consumption, antibiotic prescribing trends

## Abstract

Background and objective: The increasing emergence and spread of drug-resistant pathogens resulting from inappropriate antibiotic usage have become more evident in recent years, particularly with the rising incidence of methicillin-resistant Staphylococcus aureus (MRSA) infections. Since joining the Organization for Economic Cooperation and Development (OECD), Costa Rica can now compare its healthcare system with other countries, and similarities have been noted with Italy regarding health indicators. Both nations have universal healthcare systems, covering their entire populations, and hold similar positions in the Human Development Index (HDI). Consequently, the goal is to compare antibiotic prescribing and consumption patterns to collaboratively develop strategies against bacterial resistance.

Methods: In order to compare antibiotic consumption between regions, a standardized contrast was utilized, specifically using the defined daily dose (DDD). An Orthogonal Contrast test was performed to test the means, followed by the application of the Student's t-test on these contrasts. This analysis aimed to assess the potential influence of regions on DDD values. Antibiotic consumption data were collected between January 2021 and December 2022 from the Local Health Authority of Naples 3 South (LHANS) in Italy and IMS Health, Q Quintiles, and VIA by way of (IQVIA) reports in Costa Rica.

Results: LHANS shows a considerable disparity in gross expenditure compared to Italy's overall expenditure, while the private sector of Costa Rica exhibits even lower gross expenditure than Italy. Antibiotic consumption in Italy exceeds that of Costa Rica, with Costa Rica's consumption amounting to 47.70% of Italy's total consumption. Additionally, LHANS exhibited a 22.43% higher gross expenditure compared to the Campania region, emphasizing the variability in antibiotic usage within the same country The results indicated no statistically significant differences in antibiotic consumption between the regions, as none of the null hypotheses were rejected.

Conclusions: The study provides valuable insights into expenditure patterns and antibiotic consumption, highlighting the need for improved prescribing practices and awareness campaigns to address the issue of antibiotic resistance. The findings emphasize the importance of implementing international guidelines to combat the growing threat of antibiotic resistance and ensure the effective management of infectious diseases.

## Introduction

The spread of drug-resistant pathogens has only become apparent in recent years. In the last three decades, improper or discontinuous intake of antibiotics has led to the emergence of antibiotic-resistant microorganisms. The overuse and misuse of antibiotics are the main causes of this critical issue, as bacteria have had the chance to adapt and overcome the drugs effective for bacterial infections [1-3]. Antibiotic resistance can lead to longer hospital stays, higher medical costs, and often death [4]. Since Costa Rica became a member of the Organization for Economic Cooperation and Development (OECD), it has become possible to compare its healthcare system with other countries. Similarities have been observed between Costa Rica and Italy in terms of health indicators. Both nations have universal healthcare systems aiming to provide coverage for their entire populations, and they also share similar positions in the Human Development Index (HDI). Therefore, the intention is to compare patterns of antibiotic prescribing and consumption in order to develop collaborative strategies to combat bacterial resistance [5,6]. According to a report from the Costa Rican Institute for Research and Education in Nutrition and Health in 2018 (INCIESA, by its acronym in Spanish), a total of 303 methicillin-resistant Staphylococcus aureus (MRSA) isolates were obtained, and these could be classified as 59.0% associated with healthcare and 40.9% associated with the community [7]. Moreover, the scarce development of new antibiotic agents makes it crucial to address proper measures aimed at administering antibiotics responsibly to tackle this critical issue [8].

Nowadays, antimicrobial resistance (AMR) represents a global health threat and makes bacterial infections difficult to treat. In 2019, AMR was associated with over 1.27 million deaths worldwide [9]. By 2050, without prompt intervention, it is estimated that up to 10 million deaths per year will be caused by bacterial infections [10,11]. AMR can affect people of all different ages and has an alarming impact on public healthcare, as well as on veterinary and agriculture fields [12]. AMR infections that require more lines of treatment can impair patients' health due to the onset of severe side effects, prolonging care and recovery. In fact, several medical procedures or treatments rely on the ability to prevent infections, such as organ transplants, cancer therapy, and many treatments for chronic diseases like rheumatoid arthritis or diabetes, which require the administration of antibiotics [13,14].

In addition, AMR infections can raise the risk of developing new health threats such as pandemics [15-17]. When antibiotics are misused or overused, bacterial pathogens become resistant to them. As a result, when a patient is infected by a multidrug-resistant bacterium, antibiotics no longer work. Recently, new resistance is developing with rapid spread, making the treatment of many infectious diseases critical, such as sepsis, gonorrhea, pneumonia, and tuberculosis, among others [18,19]. This critical issue is exacerbated in specific areas of the world where antibiotics are taken without a prescription or in clinical contexts where guidelines are not properly applied [20]. Without immediate and proper action, there is a risk of entering a post-antibiotic era in which even a minor infection will have severe consequences [19]. Antimicrobial stewardship program plays an important role in improving the appropriate use of carbapenem and combatting AMR [21].

By 2025, a decade would have elapsed since the implementation of the World Health Organization (WHO) Global Plan of Action (GPA) to combat AMR, in which five strategic objectives are proposed to reduce this global problem [12,20,22]. One of the planned objectives is to promote the optimal use of antimicrobials through Antimicrobial Stewardship Programs, these programs have been implemented in many countries, demonstrating improved patient outcomes, and decreased AMR and healthcare-associated infections [23].

In Costa Rica, these programs have been shown to improve the consumption of antibiotics such as first-generation cephalosporins, third-generation cephalosporins, and fluoroquinolones, as well as changes in the prescription habits of medical specialists in surgery and intensive care [24-26]. On the other hand, in Italy, the implementation of these programs has improved patient clinical outcomes in terms of readmission, mortality rate, improvements in therapy selection, and cost reduction [27]. Despite certain advancements, there is a pressing need to undertake more impactful and tangible measures to address this issue [20,22,28]. Therefore, it is crucial to address this phenomenon through a Global Health approach, which is multidisciplinary and characterized by a holistic view that integrates resources in the environmental, human, and veterinary fields [28]. Italy is one of the European countries with the highest levels of AMR, which result from high and often inappropriate antibiotic use [29]. Similarly, the antibiotic resistance issue is also emerging in Costa Rica, being a global emergency, which involves all clinical settings worldwide [29]. The calculation of the defined daily dose (DDD) has been instrumental in analyzing antibiotic consumption. The DDD serves as a global benchmark for determining the average daily dosage of a drug relative to its therapeutic indication. By utilizing this indicator, researchers can evaluate the level of exposure or therapeutic intensity within a specific population, making it easier to compare data across diverse time periods and different groups of individuals. This standardized approach not only enables meaningful comparisons but also allows for the assessment of prescribing practices, identification of variations in antibiotic usage patterns, and evaluation of interventions aimed at optimizing antibiotic utilization. The DDD is an invaluable tool for monitoring trends in antibiotic consumption and facilitating evidence-based strategies to address AMR [29].

The objective of this study is to evaluate the prescribing trends of antibiotics in two countries that differ in geographic area and socioeconomic development to contrast the data collected. A direct comparison of data from a Western Europe country with one in Latin America can provide new and important evidence, useful in enriching the existing literature since lower respiratory infections are the leading causes of death in both income groups [29,30].

## Materials and methods

Study design

This is a retrospective comparative analysis, conducted between January 2021 and December 2022, of antibiotic consumption between Italy and Costa Rica. In Italy, the search has been conducted via the Local Health Authority of Naples 3 South (LHANS), an authority characterized by providing healthcare to a population of over one million inhabitants, which comprehends 10 health districts and five hospitals. In Costa Rica, the study has been conducted by extracting information from the IQVIA reports (IMS Health, Q Quintiles, and VIA by way of), an American multinational company serving as a global provider of healthcare data and analytics, and their reports are highly relevant when it comes to understanding drug consumption trends per country. IQVIA collects and analyzes vast amounts of data from various sources, including pharmacies, hospitals, and healthcare providers, to provide comprehensive and representative insights into the pharmaceutical market. The research study strictly adhered to the established guidelines known as Strengthening the Reporting of Observational Studies in Epidemiology (STROBE). These guidelines ensure comprehensive and transparent reporting of observational studies, enhancing the overall quality and credibility of the research findings.

Data collection

Data compilation for this study involved accessing specific sources to gather comprehensive information on drug consumption in different countries. For instance, in Italy, the “Tessera Sanitaria” (TS) database was consulted to analyze all antibiotic prescriptions and dispensations during the studied period in the LHANS and Campania Region. The TS database, a national healthcare system, served as a valuable resource by storing healthcare data for the Italian population.

Similarly, in Costa Rica, IQVIA collects data from multiple sources, including pharmacies, hospitals, and healthcare providers. Additionally, they may obtain data on drug sales and prescriptions directly from pharmaceutical companies. By leveraging this diverse range of data sources, IQVIA can generate representative insights into drug consumption patterns in the private sector of this country.

Consumption

Consumption has been analyzed by calculating the DDD of the antibiotics used in the studied period, the calculations were conducted using consumption reports obtained directly from the mentioned databases to create tables to compare regions and countries. The analysis of drug costs involved expressing them in gross euros as gross expenditure multiplied by 1,000 inhabitants/day. To ensure comparability, the drug prices in Costa Rica were converted to net euros using the current exchange rate in May 2023.

Sales or prescription data expressed as DDDs per 1,000 inhabitants per day can offer an approximate estimation of the proportion of the study population receiving daily treatment with a specific drug or group of drugs. For instance, a value of 10 DDDs per 1,000 inhabitants per day signifies that, on average, in a representative group of 1,000 individuals, 10 DDDs of the drug are used on any given day within the analyzed year.

Data analysis

The data were collected, classified, and analyzed using Microsoft® Excel® for Microsoft 365 MSO (Microsoft, Redmond, Washington, DC, USA) and IBM® SPSS Statistics version 28 (IBM, Chicago, IL, USA). Descriptive statistics, such as frequencies and percentages, were calculated.

Subsequently, a standardized antibiotics consumption comparison between Italy and Costa Rica was conducted using an Orthogonal Contrast test, broken down by sectors [31]. This statistical analysis tool is highly useful for examining experimental data, facilitating mean comparisons between diverse data groups, and obtaining specific residuals. In this case, it is useful to assess the impact of data from various regions on the average antibiotic consumption. This analysis was carried out using the data processing program R version 4.3.0.

The following hypotheses are proposed. H0: The effect on the mean DDD of CR is the same as the others vs H1: The effect on the mean DDD of CR is smaller than the others. Then, H0: The effect on Italy's average DDD is equal to that of its region’s vs H1: The effect on the mean DDD of LHANS is smaller than that of the other regions. Finally, H0: The effect on the mean DDD of Campania is equal to that of LHANS vs H1: The effect on the mean DDD of Campania is less than that of LHANS to formulate a Student's t-test to the contrasts.

## Results

Tables 1, 2 present the comparative data on gross expenditure and antibiotic drug usage for Italy, the Campania Region, LHANS, and the private sector of Costa Rica. LHANS demonstrates notably higher gross expenditure compared to Italy's overall expenditure, whereas the private sector of Costa Rica exhibits even lower gross expenditure than Italy. Antibiotic consumption in Italy surpasses that of Costa Rica, with Costa Rica's consumption amounting to 47.70% of Italy's total consumption.

**Table 1:** Gross expenditure on antibacterial drugs (ATC J01 Classification) comparison between Italy, Campania Region, LHANS, and private sector of Costa Rica. ATC: Anatomical Therapeutic Chemical code, LHANS: Local Health Authority of Naples 3 South

Population studied	ATC J01 Gross expenditure (€) x 1,000 inhabitants/day
Italy	24.99
Campania	42.35
LHANS	51.85
Costa Rica Private Sector	11.92
Comparison	Change %
LHANS vs Italy	107.48
LHANS vs Campania	22.43
LHANS vs Costa Rica	334.98

**Table 2:** Antibacterial drugs (ATC J01 Classification) consumption between Italy, Campania Region, LHANS, and private sector of Costa Rica. ATC: Anatomical Therapeutic Chemical code, LHANS: Local Health Authority of Naples 3 South

Population Studied	ATC J01 DDD 1,000 inhabitants/day
Italy	14.30
Campania	19.40
LHANS	22.58
Costa Rica Private Sector	6.33
Comparison	Change %
LHANS vs Italy	57.84
LHANS vs Campania	16.40
LHANS vs Costa Rica	256.31

Compared to Italy, LHANS experienced a significant 107% change in antibiotic expenditure demonstrating a substantial difference between these regions. Furthermore, LHANS exhibited a 22.43% higher gross expenditure compared to the Campania region, underscoring the variability in antibiotic cost within the same country.

Table 3 presents data on antibiotic consumption in LHANS and Costa Rica. LHANS demonstrates a significantly higher level of antibiotic usage compared to Costa Rica, as evidenced by the discrepancy in gross expenditure. Moreover, the expenditure per DDD is also higher in LHANS, indicating that the increase in gross expenditure surpasses the proportional increase in DDD.

**Table 3:** Antibacterial drugs (ATC J01 Classification) spending and consumption in year 2022 vs year 2021 in Campania Region, LHANS, and private sector of Costa Rica. ATC: Anatomical Therapeutic Chemical code, LHANS: Local Health Authority of Naples 3 South

Population Studied	ATC J01 Gross expenditure (€) x 1000 inhabitants/day	Change % 2022 *vs* 2021	LHANS *vs* Campania Region	DDD 1000 inhabitants/day	Change % 2022* vs* 2021	LHANS *vs* Campania Region
LHANS	51.85	11.55%	36.63%	22.58	17.67%	36.65%
Costa Rica Private Sector	12.72	33.09%	-	6.74	24.20%	-

Table 4 shows a comparative analysis of antibiotic usage and gross expenditure in LHANS for the year 2022 compared to 2021, including the 20 most frequently used antibiotics in this region. The data reveal notable changes in the consumption of various antibiotics and the associated costs.

**Table 4:** LHANS - top 20 active ingredients in descending order of consumption - 2022 vs 2021. DDD: Defined Daily Dose

Description	LHANS		Change % 2022 vs 2021	
	DDDx1000 inhabitants/day	Gross expenditure x 1000 inhabitants/day	DDD x 1000 inhabitants/day	Gross expenditure x 1000 inhabitants/day
Amoxicillin	0.85	0.53	5.5%	4.6%
Amoxicillin and beta-lactamase inhibitor	7.23	11.22	22.1%	20.1%
Azithromycin	3.39	5.37	10.8%	3.5%
Cefaclor	0.05	0.10	29.8%	24.4%
Cefditoren	0.56	2.18	- 7.9 %	- 73.6 %
Cefixime	1.68	4.39	44.9%	44.8%
Ceftazidime	0.06	1.22	5.1%	6.6%
Ceftriaxone	0.66	8.83	8.2%	6.7%
Cefuroxime	0.06	0.09	- 3.3 %	- 3.4 %
Ciprofloxacin	1.46	4.16	6.0 %	5.1%
Clarithromycin	3.18	3.29	49.5%	51.3%
Doxycycline	0.17	0.07	- 0.7 %	- 1.2 %
Fosfomycin	0.54	2.80	0.3%	- 0.6 %
Levofloxacin	1.15	1.86	5.4%	1.6%
Limecycline	0.14	0.13	- 13.3 %	- 13.8 %
Minocycline	0.07	0.09	- 3.7 %	- 4.3 %
Nitrofurantoin	0.28	0.28	- 0.9 %	- 1.9 %
Prulifloxacin	0.12	0.53	- 10.9 %	- 13.6 %
Roxithromycin	0.16	0.43	47.6%	46.8%
Sulfamethoxazol and trimethoprim	0.39	0.20	0.1%	- 1.3 %

The gross expenditure for antibiotic usage showed a mixed pattern, with both increases and decreases observed. While some antibiotics demonstrated a rise in associated costs, including clarithromycin, cefixime, and ceftazidime, others experienced reductions in expenditure, such as cefditoren, fosfomycin, and sulfamethoxazole and trimethoprim.

Table 5 lists the 20 principal antibiotics in the private sector of Costa Rica, revealing differences not only in the top three agents but also in the overall availability of antibiotics. Among the 20 antibiotics listed in LHANS, cefditoren, roxithromycin, lymecycline, prulifloxacin, minocycline, and cefaclor are not available in Costa Rica. Azithromycin, doxycycline, and amoxicillin with a beta-lactamase inhibitor are the most used antibiotics in the private sector of Costa Rica, accounting for 13.12% of the total DDD for this region. In terms of gross expenditure, the three agents with the highest values were azithromycin, clarithromycin, and amoxicillin with a beta-lactamase inhibitor. In comparison with LHANS, the gross expenditure values in the private sector of Costa Rica do not correlate with DDD, as the cost of antibiotics with epidemiological impact is usually higher.

**Table 5:** Costa Rica's private sector - top 20 active ingredients in descending order of consumption - 2022 vs 2021. DDD: Defined Daily Dose

Antibiotic	Costa Rica private sector		Change % year 2022 vs year 2021	
	DDD x 1,000 inhabitants/day	Gross expenditure x 1,000 inhabitants/day	DDD x 1,000 €/day	Gross expenditure x 1,000 €/day
Amoxicillin	0.320	17.272	17.272	15.600
Amoxicillin and beta-lactamase inhibitor	0.904	20.938	20.938	21.066
Ampicillin	0.005	-0.412	-0.412	-0.534
Azithromycin	1.899	34.275	34.275	32.484
Aztreonam	0.005	-0.517	-0.517	-0.541%
Cefadroxil	0.051	1.312	1.312	1.355
Cefixime	0.153	13.069	13.069	12.654
Ceftriaxone	0.098	1.534	1.534	1.034
Cefuroxime	0.059	-0.636	-0.636	-0.750
Cephalexin	0.043	-0.117	-0.117 %	-1.347
Ciprofloxacino	0.176	-4.123	-4.123 %	-4.638
Clarithromycin	0.144	21.781	21.781	19.373
Clindamycin	0.018	-0.581	-0.581	-0.651
Doxycycline	1.791	0.899	0.899	1.003
Ertapenem	0.007	0.070	0.070	-0.026
Levofloxacin	0.494	11.389	11.389	7.730
Ofloxacin	0.029	0.030	0.030	0.009
Penicillin	0.004	-0.212	-0.212	-0.380
Sulfamethoxazole and trimethoprim	0.091	-0.885	-0.885	-0.801
Tetracycline	0.024	-0.148	-0.148	-0.158

Additionally, as shown in Table 6, the comparison between Italy and the private sector of Costa Rica shows that overall antibiotic consumption in Italy is significantly higher than in the private sector of Costa Rica, as expected. An important consideration to consider in this comparison of DDD is the notable difference between LHANS, Campania, and Italy, with the highest consumption observed in LHANS. This highlights the need to establish more stringent regulations for antibiotic prescriptions in the LHANS region.

**Table 6:** Comparison of antibiotic consumption between Italy and private sector of Costa Rica. DDD: Defined Daily Dose, ND: No data

Description	Italy	Campania	LHANS	CR private sector
	DDD x 1,000 inhabitants/day	DDD x 1,000 inhabitants/day	DDD x 1,000 inhabitants/day	DDD x 1,000 inhabitants/day
Amoxicillin	0.85	0.86	0.85	0.32
Amoxicillin and beta-lactamase inhibitor	5.19	6.38	7.23	0.32
Azithromycin	1.79	2.76	3.39	1.90
Cefaclor	0.04	0.05	0.05	ND
Cefditoren	0.24	0.37	0.56	ND
Cefixime	1.16	1.45	1.68	0.15
Ceftazidime	0.01	0.04	0.06	0.0004
Ceftriaxone	0.21	0.53	0.66	0.10
Cefuroxime	0.08	0.08	0.06	0.06
Ciprofloxacin	0.67	1.19	1.46	0.18
Clarithromycin	1.77	2.65	3.18	0.14
Doxycycline	0.18	0.16	0.17	1.79
Fosfomycin	0.38	0.47	0.54	NI
Levofloxacin	0.72	1.07	1.15	0.49
Limecycline	0.11	0.14	0.14	ND
Minocycline	0.04	0.06	0.07	NI
Nitrofurantoin	0.15	0.21	0.28	NI
Prulifloxacin	0.07	0.08	0.12	ND
Roxithromycin	0.04	0.12	0.16	ND
Sulfamethoxazole and trimethoprim	0.34	0.38	0.39	0.09

Finally, the Student's t-test applied to the contrasts resulted in the following p-values; H1: p=0.8475, H2: p=0.8023, and H3: p=0.6382. None of the hypotheses raised is rejected, so it is concluded that the region has no statistically significant effect on DDD.

## Discussion

Gross expenditure

Firstly, the analysis reveals a difference in gross expenditure (Table 1) between LHANS and the total expenditure for Italy. The higher gross expenditure in LHANS suggests increased healthcare costs associated with antibiotic acquisition and administration in this region. This could be attributed to factors such as a higher prevalence of infections, the negative effect of the COVID-19 pandemic, greater reliance on antimicrobial treatments, or even differences in healthcare policies and practices [15]. The notable 107% surge in LHANS' antibiotic consumption in comparison to Italy underscores the urgency for enhanced monitoring and intervention to promote judicious antibiotic use. Excessive consumption of antibiotics is widely recognized as a significant factor in the rise of antibiotic resistance, emphasizing the importance of taking proactive measures to address this issue [19].

Moreover, the comparison between LHANS and the Campania Region reveals variations in both gross expenditure and antibiotic consumption. The 22.43% higher gross expenditure in LHANS indicates differences in healthcare resource utilization, emphasizing the importance of studying regional variations within a country [32,33]. These findings also underscore the need for targeted interventions at the regional level to promote responsible antibiotic prescribing and optimize healthcare resources, as seen in other studies from the same country [15,34,35].

In contrast, the private sector of Costa Rica exhibits lower gross expenditure compared to Italy's total. This indicates potential cost-effectiveness in the private healthcare system of Costa Rica, suggesting the presence of measures to control healthcare spending [36]. However, it is important to note that lower expenditure does not necessarily imply lower antibiotic consumption. The analysis reveals that antibiotic consumption in Costa Rica represents 47.70% of the total consumption in Italy, indicating differences in prescribing practices and healthcare utilization between the two countries. Costa Rica's expenditure on medicines represents 8.2% of the country's total healthcare spending. Within this, the consumption of antibiotics in the private market accounts for 2.21%, which amounts to a significant expense [37-39].

Antibiotic consumption

The comparison between LHANS and Costa Rica shown in Table 2 also reveals differences in antibiotic consumption and gross expenditure. LHANS exhibits a tendency for higher consumption and gross expenditure compared to Costa Rica's private sector [37,38].

Costa Rica has evidenced a significant improvement in antibiotic prescription practices since the implementation of the Stewardship Programs in an inpatient setting. This program, like initiatives in other countries, focuses on developing internal prescription protocols for the most common infectious diseases at the local level in various hospitals [40]. Its primary objective is to optimize the utilization of available antibiotics within the country and mitigate the development of AMR. These efforts have been clearly reflected in the prescription patterns of antibiotics in this locality in recent years [36,41].

Comparison of antibacterial drug expenditure and consumption

The temporal analysis of antibiotic consumption and gross expenditure shown in Table 3, within LHANS, reveals notable changes in the utilization of specific antibiotics and associated costs. Increases in the usage of certain antibiotics suggest a potential need for monitoring their appropriate use to prevent overprescribing and minimize the risk of antibiotic resistance [40,42]. Conversely, reductions in the consumption of other antibiotics may be indicative of evolving treatment guidelines or the availability of alternative treatment options. As mentioned, the mixed pattern of changes in gross expenditure emphasizes the dynamic nature of healthcare costs, influenced by factors such as drug pricing, prescribing practices, and healthcare policies within the country [38,41-43].

LHANS experienced a significant increase in gross expenditure and DDD in comparison with Campania. This suggests a higher prevalence of antibiotic prescriptions or an increased dependency on antimicrobial treatments within LHANS. These findings raise concerns about the potential overuse of antibiotics within the region. It highlights the need for closer monitoring and appropriate interventions to promote responsible antibiotic prescribing practices and combat the growing issue of antibiotic resistance.

Tables 4, 5 provide insights into the most frequently used active ingredients, while Table 6 presents a comparative analysis. Among antibiotics, notable increases in usage were observed for amoxicillin and beta-lactamase inhibitors, azithromycin, clarithromycin, cefixime, and ciprofloxacin. Conversely, several antibiotics experienced a decline in consumption, including cefditoren, lymecycline, prulifloxacin, and minocycline. These reductions may indicate a decrease in prescription rates, possibly due to alternative treatment options or evolving treatment guidelines.

Notably, ceftriaxone generated a substantial total expenditure of 8.83, despite having a DDD of only 0.66. This emphasizes the importance of considering expenditure variations when selecting antibiotics in the region, as factors such as changes in drug pricing, prescribing patterns, and healthcare policies can influence these variations [44].

In terms of changes between 2021 and 2022, ciprofloxacin usage decreased, while there was an increase in the usage of azithromycin, amoxicillin, and amoxicillin with beta-lactamase inhibitors. These changes align with the implementation of antibiotic stewardship in ambulatory prescriptions indicating a positive impact on prescribing practices in Costa Rica [41].

A notable difference to highlight is the consumption of doxycycline, with the private sector of Costa Rica exhibiting the highest consumption rate (1.79 DDD per 1,000 inhabitants per day), followed by Italy (0.18 DDD per 1,000 inhabitants per day), LHANS (0.17 DDD per 1,000 inhabitants per day), and Campania (0.16 DDD per 1,000 inhabitants per day).

Additionally, the study emphasizes the importance of implementing ambulatory antibiotic stewardship programs. These programs play a crucial role in promoting responsible antibiotic use, minimizing unnecessary costs, and addressing the emerging challenge of antibiotic resistance. By involving pharmacists in the process, these programs can leverage their expertise to optimize prescribing practices, educate patients and healthcare providers about appropriate antibiotic use, and monitor the outcomes of antibiotic therapies. This collaborative approach can enhance the effectiveness of interventions, ensure patient safety, and improve overall healthcare outcomes in the studied regions and countries [44].

**Figure 1:**
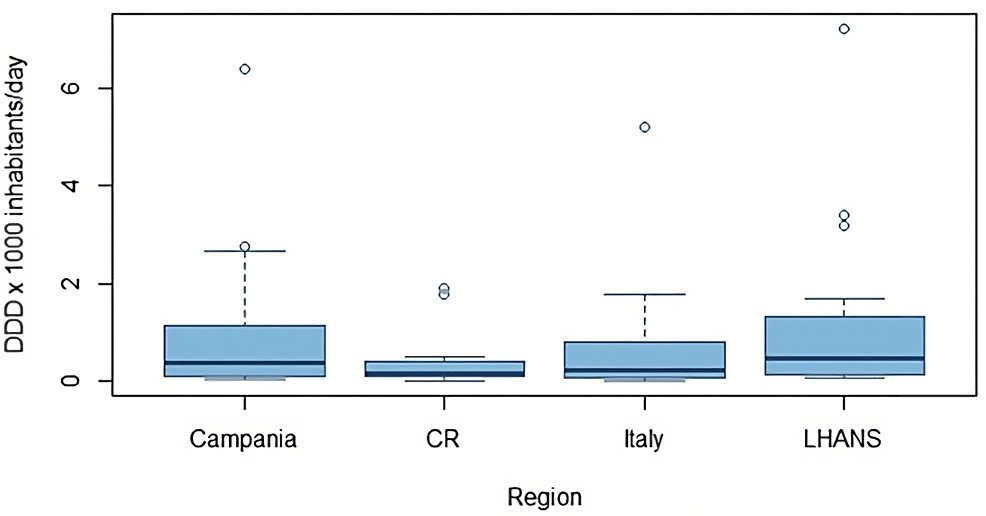
Comparative consumption distribution average across regions in Italy and Costa Rica. DDD: Defined Daily Dose, CR: Costa Rica, LHANS: Local Health Authority of Naples 3 South *In these plots, most of the data is located within the boxes, the lines represent data that deviates from the average for that region, and the dots represent extreme values. In this case, they represent active components with very high DDDs compared to the others.

Based on the results presented in Table 6 and graphically represented in Figure 1, it is evident that Costa Rica shows a downward trend in average consumption relative to Italy and its studied regions. Based on the above, an orthogonal contrast test was proposed to evaluate if there are statistical differences in the influence that regions have on the mean consumption of antibiotics in each case, using the average standardization of data (DDD). When performing the Student’s t-test on the contrasts with the hypotheses proposed in the methodology, the p-values indicate that there is no difference between the means of average antibiotic consumption between regions. This leads to the conclusion that the analyzed regions do not have an influence on antibiotic consumption. It is important to highlight that despite the graphical trend of a decrease in consumption in Costa Rica shown in Figure 1, the differences in means are not statistically significant, thus emphasizing the need for biostatistical tests in this type of study.

This direct comparison between Italy and Costa Rica provides novel evidence, considering their different geographic and socioeconomic backgrounds. As lower respiratory infections are the leading causes of death worldwide, the findings have relevance in addressing this global health issue. The study's methodology, utilizing national health databases and reputable sources, ensures reliable and representative data. Calculation of DDD and analysis of drug costs contribute valuable insights into consumption patterns and economic aspects [41].

The results of this comparative study provide valuable insights into the patterns of gross expenditure and antibiotic consumption between two very different countries: Italy and Costa Rica. Understanding these variations can be helpful in identifying potential areas of improvement in each country and promoting the development of more responsible antibiotic prescribing practices.

## Conclusions

As illustrated in the results, when compared to 2021, the trend of antibiotic consumption in 2022 in both countries exhibited a steady rise, resulting in increased pharmaceutical expenditure and potentially contributing to the antibiotic resistance phenomenon. Although global campaigns aimed at raising awareness and reducing the use of antimicrobial agents have been conducted, they have not yielded the anticipated outcomes. The recent global action plan advocated by the WHO aims to address the issue of antibiotic prescriptions across all healthcare fields.

To raise awareness among healthcare professionals and all stakeholders regarding the issue of antibiotic resistance, it is crucial for health authorities to develop information campaigns. These campaigns should aim to educate all stakeholders about the harmful effects of improper antibiotic use. Without access to effective alternative treatments, various projections indicate that the health risks associated with infectious diseases will increase. This retrospective analysis, conducted in two distinct geographical areas, underscores the need to address the issue of antibiotic resistance by sharing and implementing international guidelines.
